# Unraveling the key mechanisms of *Gastrodia elata* continuous cropping obstacles: soil bacteria *Massilia*, *Burkholderia-Caballeronia-Paraburkholderia*, and *Dyella* along with soil metabolites 4-hydroxy-benzenemethanol and N-(2-butyl)-N-octadecyl-, ethyl ester as crucial indicators

**DOI:** 10.3389/fmicb.2024.1478330

**Published:** 2024-10-28

**Authors:** Mingzheng Duan, Chengcui Yang, Liuyuan Bao, Duo Han, Haiyan He, Yongzhi Zhang, Li Dong, Shunqiang Yang

**Affiliations:** ^1^Yunnan Key Laboratory of Gastrodia and Fungi Symbiotic Biology, Zhaotong University, Zhaotong, China; ^2^Yunnan Engineering Research Center of Green Planting and Processing of Gastrodia, Zhaotong University, Zhaotong, China; ^3^College of Agronomy and Life Sciences, Zhaotong University, Zhaotong, China

**Keywords:** *Gastrodia elata*, continuous cropping obstacles, soil, 16S rRNA metabarcoding, metabolomics

## Abstract

**Background:**

Tian-ma (*Gastrodia elata*) is a traditional medicinal herb found in China. It is used in healthy food and to treat various diseases, therefore cultivated extensively in southwest China. However, continuous cropping of this species has led to various obstacles, such as microbial disease and pest infestation, significantly affecting the production and development of valuable medicinal and food resources. As per the growth habit, soil is presumed to be the primary factor contributing to these obstacles, despite the known issues of continuous cropping obstacles in *Gastrodia elata*, such as microbial disease, there is a lack of comprehensive understanding of the specific soil bacterial communities and metabolites involved in these processes.

**Methods:**

We analyzed soil samples collected during the year of Tian-ma cultivation (0 Year), after the Tian-ma harvest (1 Year), after two years (2 Year), and three years (3 Year) of fallowing post-cultivation using soil 16S rRNA metabarcoding sequencing by illumina platform and metabolomics (GC–MS/MS). Soil sample collected from the uncultivated field was used as the control (CK).

**Results:**

Metabarcoding sequencing showed high bacterial alpha diversity during the cultivation of Tian-ma (0 Year) and the period of deterioration of soil bacterial community. (1 Year), with decreased anaerobic bacterial abundance and increased copiotrophic bacterial abundance. Bacteria associated with sulfur metabolism also showed increased abundance during the year of cropping obstacles. Further metabolomics approach identified 4-hydroxy-benzenemethanol as an indicator of Tian-ma continuous cropping obstacles. Besides, metabolites of the carbohydrate class were found to be the most abundant during the occurrence of continuous cropping obstacles of *Gastrodia elata*, suggesting that regulation of soil microbial diversity may be a critical factor in addressing these obstacles. Finally, the correlation analysis indicated a positive association between the abundance of some metabolite, e.g., carbamic acid, N-(2-butyl)-N-octadecyl-, ethyl ester detected after Tian-ma cultivation and the abundance of bacteria capable of degrading toxic metabolites, such as *Massilia*, *Burkholderia-Caballeronia-Paraburkholderia*, and *Dyella*.

**Conclusion:**

This study has revealed the specific soil bacteria and metabolic factors related to the continuous cropping obstacles of *Gastrodia elata*. These findings not only deepen our understanding of the continuous cropping issues but also pave the way for developing effective strategies to overcome them.

## 1 Introduction

*Gastrodia elata* Blume, known as Tian-ma in Chinese, is a perennial herb of the Orchidaceae family and a valuable medicinal plant produced mainly in China. In China, *G. elata* is found flourishing primarily in the montane forests (altitudes ranging from 700 to 3,200 meters) of Yunnan, Shanxi, Guizhou, Sichuan, Hubei, Jilin, and Anhui provinces (Wang et al., 2019). It is a mycoheterotrophic species that depend on fungal mycelium for food (Xu et al., 2021). The tubers of *G. elata* have been traditionally used in Chinese medicine to treat various diseases and disorders, including headache, dizziness, spasms, epilepsy, stroke, and amnesia (Liu et al., 2018). Various preclinical studies have also demonstrated diverse healthy functions, including neuroprotection, learning and memory enhancement, cardioprotection, vasodilatory effects, antidepressant properties, and anticancer effects (Zhan et al., 2016; Gong et al., 2024). This medicinal species contains glycosides, phenols, and phenolic glycosides as the primary ingredients, along with sterols, their glycosides, and polysaccharides (Liu and Huang, 2017). Researchers have also identified gastrodin (Chen et al., 2015), p-hydroxybenzyl alcohol (Hsieh et al., 1997), and parishin (A, B, C, and E) in this herb (Li et al., 2019). Thus, *G. elata* is extensively utilized in clinical settings and food industries (Gong et al., 2024). Research during the past decade has revealed that *G. elata* is a mycoheterotrophic species and has contributed to the progress in cultivating mature *G. elata* tubers (Hsieh et al., 2022).

Continuous cropping obstacles refer to the negative phenomena such as severe diseases and yield reduction that occur when the same crop is cultivated repeatedly on the same land. Similar to the traditional Chinese medicinal herb *Panax notoginseng* (Sanqi) (Bao et al., 2022), continuous cultivation of *G. elata* leads to several obstacles associated with yield reduction and even complete crop failure. These obstacles occur in the form of severe pest infestations, land degradation, and yield reductions in the second year of *G. elata* cultivation. Currently, farmers prefer replacing and sterilizing the soil before replanting at the cultivation site to manage these obstacles; however, such an approach significantly limits the production of *G. elata* and hinders the development of resources, as most cultivation sites are located in mountainous areas with difficult access, greatly increasing cultivation costs.

Unlike other orchids, *G. elata* lacks flowers and leaves and cannot obtain nutrients through photosynthesis or soil due to its unique evolutionary pathway (Yuan et al., 2018). The plant relies completely on a symbiotic relationship with the fungus *Amillariella* sp. for growth (Zhang and Li, 1980). This growth habit makes its cultivation considerably different from other plants and medicinal herbs. Therefore, the main factors contributing to the obstacles associated with continuous cropping of *G. elata* may primarily involve the interactions among *G. elata*, *Amillariella* sp., and the soil, however, there are no studies discuss this. Deficiency in nutrients, accumulation of toxic metabolites, and disruptions in the microbial ecological balance of the soil are the major challenges in the continuous cultivation of crops (Ma et al., 2023). However, the obstacles in *G. elata* differ from those in traditional medicinal plants like *P. notoginseng* since it does not directly absorb nutrients from the soil. Therefore, the mechanisms underlying these obstacles remain unclear. Considering the fact that replacing soil helps overcome these obstacles, soil may serve as a primary factor related to the obstacles. Therefore, analyzing the changes in various soil factors during the occurrence of these obstacles under *G. elata* continuous cropping will provide novel and crucial insights into their mechanisms and help in reduce cultivation costs and increase yield.

Metabarcoding and metabolomics are omics technologies that help elucidate the mechanisms underlying plant–soil interactions, therefore adopted to assess the obstacles mechanism of *G. elata*. Besides, a study based on metabarcoding and metabolomics techniques in the soil samples revealed the mechanisms in soil promoting plant growth by fairy ring fungi (Duan et al., 2022). Similar techniques unraveled the interaction between the bamboo fungus *Dictyophora indusiata* and the soil in a sugarcane field, which successfully explaining its role in mitigating soil nitrogen loss (Duan et al., 2023). The above case demonstrates that using metabarcoding sequencing and metabolomics techniques can provide new insights into the mechanisms behind continuous cropping obstacles in *G. elata*.

Therefore, the present study investigated the soil metabolic and microbial mechanisms underlying the obstacles associated with the continuous cropping of *G. elata*. We conducted metabarcoding sequencing and metabolomic analysis for the soil samples collected during *G. elata* cultivation, continuous cropping, and fallow periods to identify the soil bacteria and the metabolic factors related to the occurrence of these obstacles. The study’s findings will help improve *G. elata* cultivation techniques and reduce cultivation costs.

## 2 Materials and methods

### 2.1 Materials

The present study was carried out in a mountain land of Xiaocaoba Town of Zhaotong City (latitude and longitude is 27.34 N, 103.72 E, altitude: 2100 m; Yunnan Province, China) from 2020 to 2023. The following five soil samples were analyzed in this study: (CK) control soil from an uncultivated plot; (0 Year) soil sampled after 2023 *Gastrodia elata* cultivation and harvest; (1 Year) soil collected after continuous cultivation of *G. elata* in 2022 (continuous cropping obstacles were evident, which included severe outbreaks of pests and microbial diseases.); (2 Year) soil collected from plots left unplanted (fallow) for 2 years after 2021 *G. elata* cultivation; and (3 Year) soil collected from plots left unplanted (fallow) for 3 years after 2020 *G. elata* cultivation. All treatments were cultivated from the same cultivation site. This cultivation site is located on a mountainous area, and the soil is yellow-brown soil. The *Gastrodia elata* f. glauca (Wu Tian-ma) is used for cultivation, which same as a previous study (Xu et al., 2021). The *G. elata* tubers and *Armillaria* were provided by Yunnan Senhao Industry Co., Ltd., Yunnan, China. *G. elata* is cultivated according to cultivation blocks (50 cm × 100 cm). Cultivation blocks (Supplementary Figure S1) with the same treatment are arranged laterally at intervals of 20 cm. Cultivation blocks with different treatments are cultivated at least 500 meters apart from each other. Each cultivation block is 20 cm deep. At the bottom layer, 15 kg of wood (*Cyclobalanopsis* sp.) is laid for the growth of *Armillaria*. On the upper layer of the wood, strains of *Armillaria* (400–600 g per cultivation block) and *G. elata* seed tubers (10–15 g, five per cultivation block) are laid. The top layer is covered with soil about 5–10 cm deep to keep the ground level. The cultivation time is in March of each year (2020–2023). Since *G. elata* does not draw nutrients from the soil, no fertilization is applied to the soil for all treatments. Soil samples were taken from within a radius of 5 cm around the seed tubers of *Gastrodia elata*. All samples were collected in 2023, and each treatment was applied to at least 10 cultivation sites (each site approximately 1 m × 1 m in area). Soil samples were collected from all the plots in November 2023, and for each treatment plot, samples were collected from five randomly selected points, pooled, and then divided into three biological replicates. These samples were stored in liquid nitrogen for subsequent metabarcoding and metabolomic analyses.

### 2.2 Methods

#### 2.2.1 Metabarcoding analysis

The total DNA was extracted from 3 g of soil with the HiPure Soil DNA Kit (Magen, Guangzhou, China), following the manufacturer’s protocol (Duan et al., 2022). The 16S rDNA V3-V4 region of the ribosomal RNA gene was amplified by polymerase chain reaction (PCR) with the 341F (5′-CCTACGGGNGGCWGCAG-3′) and 806R (5′-GGACTACHVGGGTATCTAAT-3′) primers to identify the soil bacteria (Guo et al., 2017). The purified amplicons obtained from the different soil samples were pooled in equimolar ratios and used for paired-end sequencing on an Illumina Novaseq 6000 platform, according to the standard protocol.

The representative operational taxonomic unit (OTU) sequence was selected, and the taxonomy was assigned to the different OTUs with the Ribosomal Database Project classifier (version 2.2), using a naïve Bayesian model (Wang et al., 2007) and based on the SILVA database (16S rRNA metabarcoding data) (Pruesse et al., 2007). All figures were generated with the R software, and the OTUs of the various groups were compared using the VennDiagram package (version 1.6.16) in R (Chen and Boutros, 2011). The alpha-diversity indices (sobs, shannon, Simpson, chao, ace, goods_coverage) were calculated at the OTU level using QIIME (version 1.9.1) (Caporaso et al., 2010). Further, principal component analysis (PCA) was conducted with the vegan package (version 2.5.3; 2022.11.9)^1^ in R software to assess the variations in bacterial OTU composition among the treatments. The UpSet plot was created using the UpSet R package (version 1.6.16) to visualize the patterns of OTUs across the various treatments (Conway et al., 2017). Linear discriminant analysis (LDA) effect size (LEfSe) analysis was executed with the LEfSe software (Segata et al., 2011), setting the LDA score threshold at 2, to evaluate. Finally, the FAPROTAX database (Functional Annotation of Prokaryotic Taxa; version 1.0) (Louca et al., 2016) was employed to predict the ecologically relevant bacterial functions.

#### 2.2.2 Soil metabolome analysis

The metabolites of the soil samples were analyzed using a previously published method (Duan et al., 2022; Duan et al., 2023). Approximately 0.5 g of the soil sample was mixed with 1 mL of methanol: isopropanol: water (3: 3: 2; v: v: v) mixture, vortexed for 3 min, subjected to ultrasound treatment for 20 min, and centrifuged at 12,000 r/min at 4°C for 3 min. The supernatant was collected and mixed with 0.020 mL of internal standard (10 μg/mL) in a sample vial (2 mL) and allowed to evaporate under nitrogen flow. The residue was freeze-dried in a lyophilizer and derivatized by mixing with 0.1 mL of methoxy-amine hydrochloride in pyridine (0.015 g/mL), followed by incubation at 37°C for 2 h. Then, 0.1 mL of bis (trimethylsilyl) trifluoroacetamide (BSTFA) containing 1% trimethylchlorosilane (TMCS) was added to the mixture, vortexed, and incubated at 37°C for 30 min. Approximately 0.2 mL of the derivatized solution was diluted to 1 mL with n-hexane, filtered through a 0.22 μm syringe filter, stored in a freezer (−20°C), and analyzed within 24 h.

Subsequently, gas chromatography–mass spectrometry (GC–MS) was adopted to profile the metabolites in the soil. The soil extract was analyzed on an Agilent 8890 gas chromatograph coupled to a 5977B mass spectrometer, and the metabolites were separated on a DB-5MS column (30 m length × 0.25 mm i.d. × 0.25 μm film thickness; J&W Scientific, USA). Approximately 1 μL of the extract was injected into the system in front inlet mode with a split ratio 5:1, and Helium was used at a 1.2 mL/min flow rate as the carrier gas. The oven was initially held at a temperature of 40°C for 1 min; the temperature was then increased to 100°C at the rate of 20°C/min, further to 300°C at the rate of 15°C/min, and finally held at 300°C for 5 min. All samples were analyzed in scan mode with the ion source and transfer line temperatures set at 230 and 280°C, respectively.

Unsupervised principal component analysis (PCA) was performed for the data scaled to unit variance before PCA using the prcomp function in R. Metabolites with variable importance in projection (VIP) ≥ 1 and an absolute log_2_ fold change (log_2_FC) ≥ 1 were considered significantly different between CK and the treatments (0 Year, 1 Year, 2 Year, or 3 Year). The VIP values were obtained from orthogonal partial least squares discriminant analysis (OPLS-DA) results, including score plots and permutation plots generated using the Metabo-Analyst R package. The original data were log_2_-transformed and mean-centered before OPLS-DA, and a permutation test (200 permutations) was performed on the data to prevent overfitting.

#### 2.2.3 Correlation analysis

The correlation between metabarcoding and metabolites was analyzed, and the heatmap based on metabarcoding and metabolites was generated using OmicShare tools, a free online platform for data analysis.^2^ The Pearson’s coefficient of correlation (R Package of psych; v.1.8.4)^3^ between the top 20 bacterial OTUs and the representative metabolites with different expression trends (K-means class) was calculated, and the significance (*p*-value) was assessed. Since the contents of various metabolites were in the same dimension, normalization was not carried out, and the original values obtained from OTUs and peak area unit were used for calculating the correlation.

## 3 Results

### 3.1 Soil bacterial diversity under continuous cropping obstacles of *Gastrodia elata*

#### 3.1.1 OTU distribution and alpha-diversity indices

**Figure 1:**
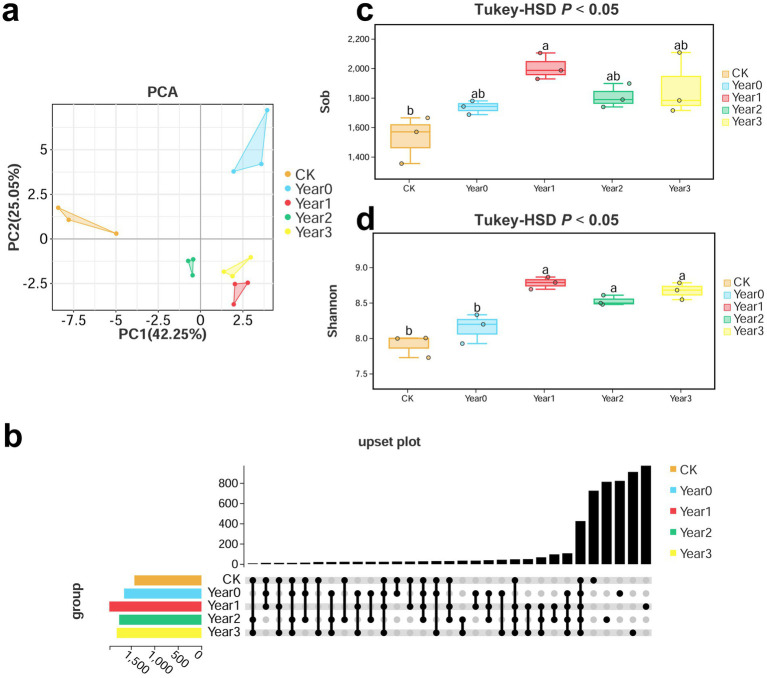
PCA, UpSet plot, and alpha-diversity indices (Sob and Shannon indices) of bacteria in soil under *Gastrodia elata* cultivation. Groups include CK, 0, 1, 2, and 3 Year (*n* = 3). (a) PCA based on bacterial OTUs. The horizontal and vertical axes of the scatter plot represent the PC1 and PC2 components, respectively. (b) UpSet plot of bacterial OTUs. The horizontal bars represent the number of species in each group. The matrix of dots in the lower right corner represents the intersection of species. Each dot represents unique information specific to a particular group, while connected dots represent shared information. (c,d) Comparison of bacterial Sob and Shannon indices. The top and bottom whiskers of the boxes indicate the maximum and minimum values, respectively; the top margin of the box indicates the upper quartile and the lower margin of the box indicates the lower quartile; the central line represents the median. The scattered points indicate the distribution of replicates within each group. Different lowercase letters indicate significant differences (*p* < 0.05).

Metabarcoding sequencing was performed using five soil samples (CK, 0, 1, 2, 3 Year) to investigate the impact of *G. elata* continuous cropping obstacles on soil bacterial diversity and its regulation. Then, we analyzed bacterial alpha-diversity based on OTUs. A High-throughput sequencing of the 16S rRNA V3-V4 region on the Illumina platform generated a total of 733,415 effective tags, with an average N90 length of 441 bp, and the number of OTUs per sample ranging from 1,354 to 2,107 (an average of 1,789 per sample) (Supplementary Table S1). PCA at OTU level showed the separation of the treatment groups (CK, 0, 1, 2, and 3 Year) and clustering of the replicates (Figure 1a). The principle components PC1 and PC2 accounted for a total of 67.3% of the variance (PC1 accounted for 42.25%, and PC2 for 25.05%). CK and 0 Year exhibited more distinct and independent clustering trends than other treatment groups (1, 2, and 3 Year), indicating that the cultivation of *G. elata* significantly regulated soil bacterial diversity. Further, the UpSet plot analysis of the distribution trends of OTUs showed that the CK, 0, 1, 2, and 3 Year had 725, 820, 951, 856, and 909 unique OTUs, respectively, and 412 shared OTUs (Figure 1b). Among the five groups, 1 Year had the highest number of unique OTUs. Subsequent evaluation of bacteria based on alpha-diversity indices showed that the sob index was significantly different only between CK and 1 Year (1,529 and 1,976; *p* < 0.05) (Figure 1c), indicating that soil bacterial diversity was most strongly disrupted in the year of continuous cropping obstacles. The average Shannon index of bacteria was significantly different between CK (7.9) and all other groups (8.1, 8.7, 8.5, and 8.6 for 0, 1, 2, and 3 Year, respectively; *p* < 0.05) and between 0 and 1 Year (*p* < 0.05; Figure 1d). The trends observed in Chao1, ACE, Simpson, and Good’s coverage indices were consistent with those in sob and Shannon indices (Supplementary Table S2). Thus, the observations on OTU distribution and alpha-diversity indices indicated that the cultivation of *G. elata* significantly affected soil bacterial diversity.

#### 3.1.2 Soil bacterial taxonomy and functions under continuous cropping obstacles of *Gastrodia elata*

**Figure 2:**
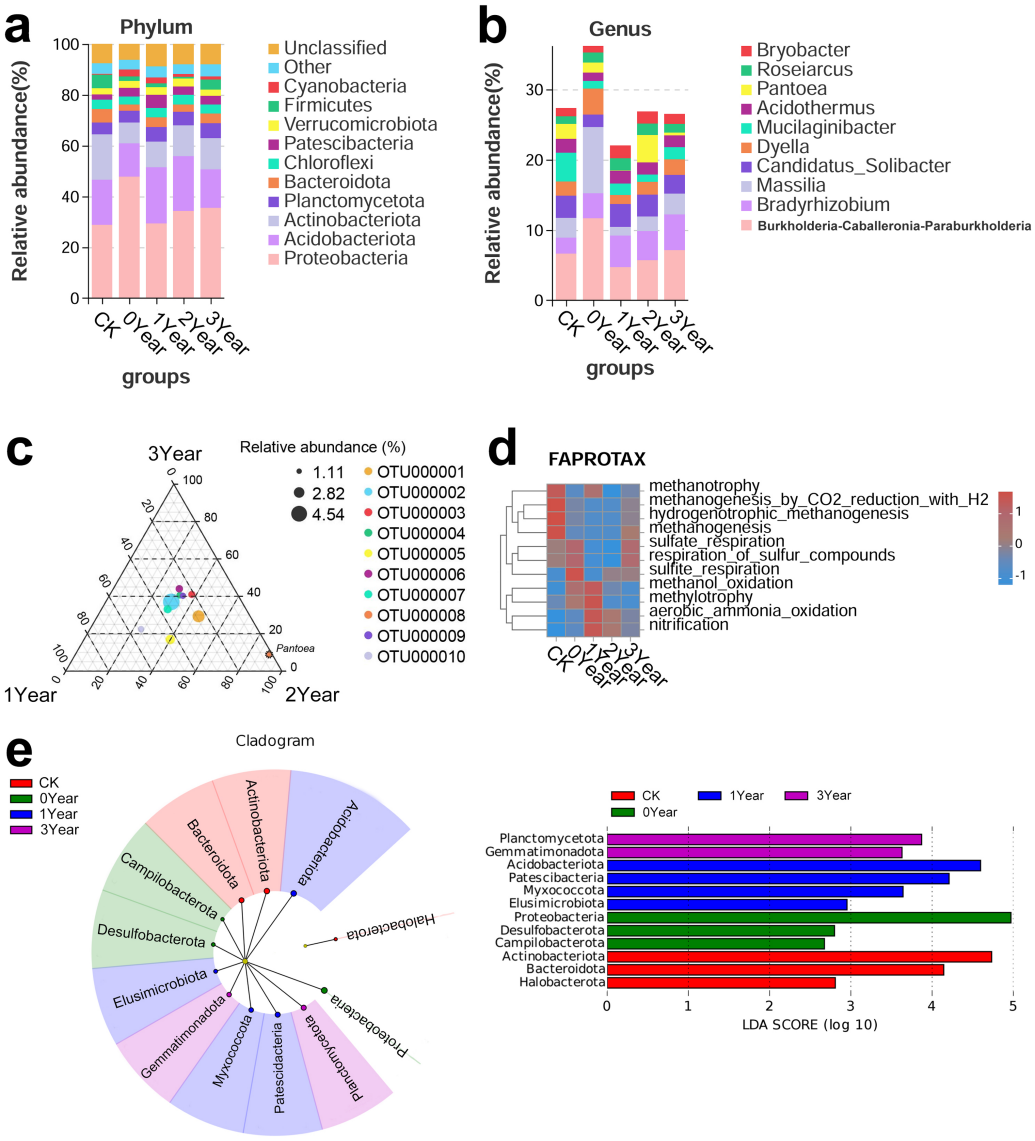
Soil bacterial taxonomy and functions. (a,b) Bar charts show the distribution of bacteria at the phylum and genus levels. The horizontal axis represents the groups, while the vertical axis represents the relative abundance of bacterial phylum or genus. (c) Ternary plot of bacterial OTUs in 1, 2, and 3 Year soil samples. Different dots represent different species, and the dot size represents the average abundance of that OTU in the three groups. The relative abundance of the species in the three groups determines the position of the dots. The dots with different colors indicate the different bacterial OTUs in which they are enriched, and the legend is shown on the right side of the subfigure. (d) Heatmap shows the annotation of bacterial OTUs based on the FAPROTAX (Functional Annotation of Prokaryotic Taxa) database. The horizontal axis represents the soil treatment groups, the vertical axis represents the bacterial functions, and the color represents the relative abundance of the bacteria with specific functions. (e) LEfSe analysis of soil bacterial taxonomy. In the figure on the left side, the concentric circles radiating from the center represent the phylum-level taxa, and the size of the sector within each circle is proportional to the relative abundance in each treatment group. The LDA score plot on the right side shows the bacterial biomarkers for different groups; here, the length of the bars represents the magnitude of the impact of the differentially abundant phylum (LDA score). The LDA scores greater than or equal to 2 are retained.

Furthermore, we investigated the impact of *G. elata* continuous cropping obstacles on soil bacterial taxonomy and functions. Taxonomic analysis based on the SILVA database revealed differences in soil bacteria among CK, 0, 1, 2, and 3 Year. Proteobacteria (28.82, 47.68, 29.29, 34.22, and 35.48% for CK, 0, 1, 2, and 3 Year, respectively), Acidobacteriota (17.72, 13.1, 22.1, 21.51, and 15.18%), Actinobacteriota (17.81, 8.27, 10.11, 12.23, and 12.27%), Planctomycetota (4.72, 4.62, 4.72, 5.26, and 5.76%), and Bacteroidota (5.3, 2.5, 3.77, 2.86, and 3.83%) were the five bacterial phyla abundant across the groups (Figure 2a). Notably, Proteobacteria exhibited significant differences in relative abundance between CK, 0 Year, and 1 Year. *Burkholderia-Caballeronia-Paraburkholderia* (6.63, 11.63, 4.69, 5.68, and 7.11% for CK, 0, 1, 2, and 3 Year, respectively), *Bradyrhizobium* (2.25, 3.58, 4.47, 4.15, and 5.06%), *Massilia* (2.81, 9.41, 1.24, 2.07, and 2.96%), *Candidatus_Solibacter* (3.15, 1.77, 3.25, 3.08, and 2.65%), and *Dyella* (2.02, 3.72, 1.32, 1.86, and 2.25%) were the most abundant genera (Figure 2b). The abundance of *Burkholderia-Caballeronia-Paraburkholderia* in 0 Year was significantly higher than in CK but substantially lower than in 1 Year. Similarly, the abundance of *Massilia* was significantly high in 0 Year. These observations indicated these genera, may involved in the process of the continuous obstacles. The detailed information on OTU Classification and abundance is shown in Supplementary Table S3.

Furthermore, ternary plots based on groups of (1, 2, 3 Year) generated to confirm the variations in bacterial abundance between the cropping year (1 Year) and the fallow years (2 and 3 Year) displayed that OTU000008 (genus *Pantoea*) dispersed between 1 and 3 Year. This observation suggested that *Pantoea* is a notable factor regulating the differences in continuous cropping obstacles and fallow process (Figure 2c).

We further analyzed the variations in soil microbial functions during the occurrence of *G. elata* continuous cropping obstacles based on the FAPROTAX database. This approach revealed that in the *G. elata* harvest year (0 Year), soil bacteria related to methane metabolism, including methanotrophy, methanogenesis by CO_2_ reduction with H_2_, hydrogenotrophic methanogenesis, and methanogenesis, significantly low. Meanwhile, the microbes involved in sulfur respiration, respiration of sulfur compounds, sulfite respiration, methanol oxidation, and methylotrophy were high (Figure 2d). The abundance of soil bacteria involved in methanol oxidation and methylotrophy rose further in the year of continuous cropping obstacle occurrence (1 Year) but returned to levels similar to CK in the fallow years (2 and 3 Year). Additionally, the abundance of aerobic microorganisms involved in ammonia oxidation and nitrification was significantly high in 1 Year but low in 2 and 3 Year.

Finally, we determined the characteristic bacterial phyla of the different soil groups based on LEfSe analysis (Figure 2e). Proteobacteria (LDA score = 4.97, *p* = 0.01), Desulfobacterota (2.79, *p* = 0.03), and Campilobacterota (2.67, *p* = 0.04) were identified as the characteristic bacterial phyla in the cultivation year (0 Year). In the year of occurrence of the continuous cropping obstacle (1 Year), Elusimicrobiota (LDA score = 2.95, *p* = 0.01), Myxococcota (3.64, *p* = 0.02), Patescibacteria (4.2, *p* = 0.03), and Acidobacteriota (4.59, *p* = 0.01) were the characteristic bacterial phyla. These observations suggested that the cultivation of *G. elata* significantly influenced soil bacteria; the diversity was further regulated during continuous cropping obstacles.

### 3.2 Soil metabolome under continuous cropping obstacles of *Gastrodia elata*

#### 3.2.1 Composition of metabolites in soil under continuous cropping obstacles of *Gastrodia elata*

**Figure 3:**
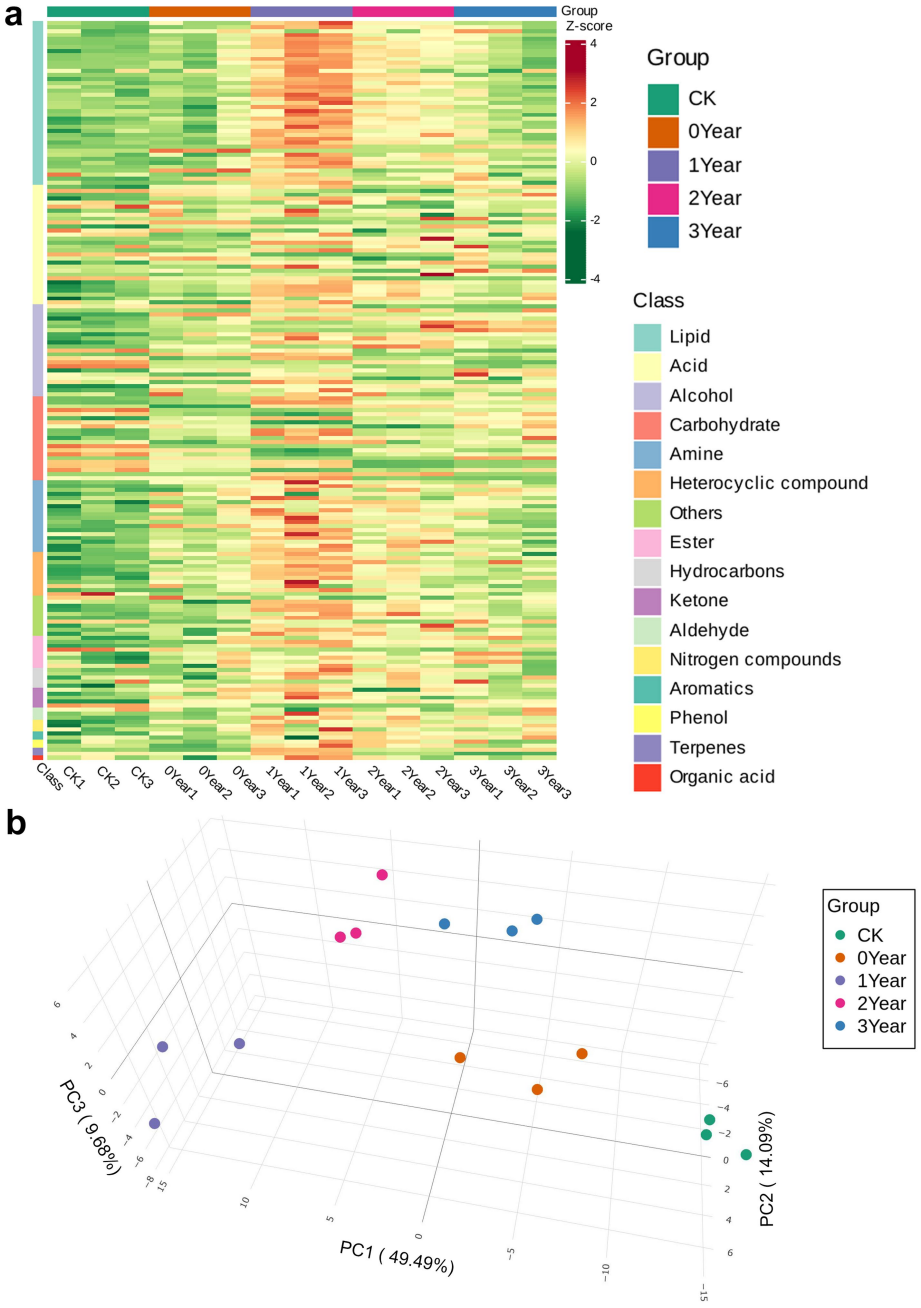
Composition of soil metabolites in soil under continuous cropping obstacles of *G. elata*. (a) Heat map of metabolites in the soil samples. Each column corresponds to a sample, and each row corresponds to a metabolite class. The color bar on the right indicates the abundance of each metabolite; here, shades of red and green indicate the upregulated and downregulated metabolites, respectively. (b) Three-dimensional PCA plot of metabolites.

We analyzed the soil metabolites associated with the occurrence of *G. elata* continuous cropping obstacles using GC–MS/MS-based soil derivatization metabolomics. The approach detected 186 metabolites, including acids (*n* = 31), alcohols (*n* = 23), aldehydes (*n* = 3), amines (*n* = 18), aromatics (*n* = 2), carbohydrates (*n* = 21), esters (*n* = 8), heterocyclic compounds (*n* = 11), hydrocarbons (*n* = 5), ketones (*n* = 5), lipids (*n* = 41), nitrogen compounds (*n* = 3), organic acids (*n* = 1), phenols (*n* = 2), terpenes (*n* = 2), and others (*n* = 10), in the five soil groups of this study. The heatmap showed that the abundance of carbohydrates in the soil in decreased trend after the cultivation of *G. elata*, while the abundance of most other metabolites, such as lipids, amines, and heterocyclic compounds, significantly increased under continuous cropping obstacles of *G. elata* during the occurrence of continuous cropping obstacles (Figure 3a). The PCA revealed the distribution trends of metabolites in the soil during the cultivation of *G. elata* and the occurrence of continuous cropping obstacles. The PC1 (49.49%), PC2 (14.09%), and PC3 (9.68%) components collectively accounted for 73.26% of the variance in metabolites (Figure 3b). The soil metabolites of the CK, 0 Year, and 1 Year samples clustered as distinct groups on the scatter plot and were arranged sequentially from −15 to +15 units along PC1, indicating a succession of metabolites in occurrence of continuous cropping obstacles among the three groups. In contrast, 2 and 3 Year appeared together but distant from the other three groups, suggesting similar metabolic processes during the two fallow years but different from the year of occurrence (1 Year) of the continuous cropping obstacles. Detailed information on metabolites and their abundance are shown in Supplementary Table S4.

#### 3.2.2 Differential metabolites in the soil under continuous cropping obstacles of *Gastrodia elata*

**Figure 4:**
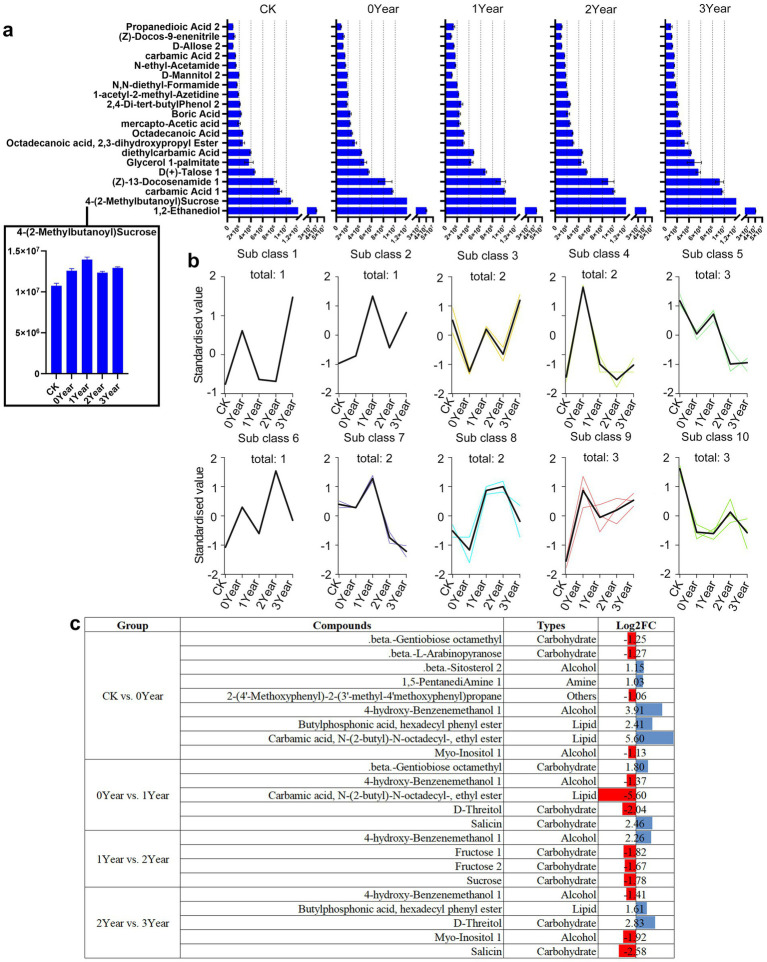
Differential metabolites in the soil during the occurrence of obstacles due to *G. elata* continuous cropping. (a) The top 20 most abundant differentially accumulated metabolites in CK, 0, 1, 2, and 3 Year. The horizontal axis indicates peak area units and the vertical axis indicates the top 20 most abundant metabolites. (b) K-means clustering of metabolites in CK, 0, 1, 2, and 3 Year. The abscissa shows the sample groups, the ordinate represents the standardized metabolite content, and the “class” represents the metabolite category number with the same trend in five groups; “total” represents the total metabolites of the specific class. (c) Differential metabolite analysis among different groups.

We further investigated the differential metabolites between the soil samples to identify the metabolic factors involved in the occurrence of obstacles after *G. elata* continuous cropping. Alcohols, carbohydrates, and acids were the main metabolic components in the all soil samples. The barchart identified 1,2-ethanediol, 4-(2-methylbutanoyl) sucrose, and carbamic acid 1 as the most abundant metabolites in the soil (Figure 4a). We then conducted K-means clustering to analyze the trends in the accumulation of metabolites across various samples. We identified a few metabolites that changed regularly with continuous cropping obstacles (Figure 4b and Table 1). For instance, two lipid metabolites of subclass 4, carbamic acid, N-(2-butyl)-N-octadecyl-, ethyl ester and hexadecanoic acid, and methyl ester, were found to be specifically accumulated in the 0 Year, the year of *G. elata* harvest. Similarly, three metabolites of subclass 5, fructose 1, fructose 2, and germanicol, were found accumulated in the soil of 1 Year, the year of occurrence of the continuous cropping obstacle, and then consistently downregulated in the second fallow year (2 Year) and the third fallow year (3 Year) (Figure 4b).

**Table 1:** Representative metabolites with different accumulation trends in the soil during the occurrence of obstacles due to *G. elata* continuous cropping.

Sub class	Compounds	Metabolite type	Formula
1	D-Threitol	Carbohydrate	C4H10O4
2	Butylphosphonic acid, hexadecyl phenyl ester	Lipid	C26H47O3P
3	2-(4′-Methoxyphenyl)-2-(3′-methyl-4′methoxyphenyl)propane	Others	C18H22O2
3	Propylene Glycol	Alcohol	C3H8O2
4	Carbamic acid, N-(2-butyl)-N-octadecyl-, ethyl ester	Lipid	C25H51NO2
4	Hexadecanoic acid, methyl ester	Lipid	C17H34O2
5	Fructose 1	Carbohydrate	C6H12O6
5	Fructose 2	Carbohydrate	C6H12O6
5	Germanicol	Alcohol	C30H50O
6	4-hydroxy-Benzenemethanol 1	Alcohol	C7H8O2
7	Squalene	Terpenes	C30H50
7	Sucrose	Carbohydrate	C12H22O11
8	beta-Gentiobiose octamethyl	Carbohydrate	C20H38O11
8	Salicin	Carbohydrate	C13H18O7
9	beta-Sitosterol 2	Alcohol	C29H50O
9	1,5-PentanediAmine 1	Amine	C5H14N2
9	2-(4-Methoxy-phenyl)-5,7-diphenyl-2,5-dihydro-pyrazolo[3,4-d]pyridazin-4-one	Ketone	C24H18N4O2
10	beta-L-Arabinopyranose	Carbohydrate	C5H10O5
10	Myo-Inositol 1	Alcohol	C6H12O6
10	bis(eta-5-piperidinylcyclopentadienyl)-Cobalt	Heterocyclic compound	C20H28CoN2

The metabolites shown in K-means clustering. The sub class see Figure 4B.

Further analysis of the differential metabolites between the adjacent comparison groups (CK vs. 0 Year; 0 Year vs. 1 Year; 1 Year vs. 2 Year; and 2 Year vs. 3 Year) revealed a significant overlap in the metabolite classes identified in the K-means clustering analysis (Figure 4c). For instance, D-threitol was upregulated by 2.83 log_2_-fold in 3 Year compared to 2 Year, but was downregulated by 2.04 log_2_-fold in 0 Year compared to 1 Year. Meanwhile, 4-hydroxy-benzenemethanol 1, a metabolite in subclass 6, exhibited a fluctuating accumulation pattern (upregulation and downregulation) during continuous cropping obstacles, e.g., it upregulated in CK compared to 0 Year and 1 Year compared to 2 Year, but downregulated in 0 Year compared to 1 Year and 2 Year compared to 3 Year. Among the 25 differential metabolites (including duplicates) of the different comparison groups, 10 (40%) were carbohydrates, including beta-gentiobiose octamethyl, beta-L-arabinopyranose, D-threitol, salicin, fructose 1, fructose 2, and sucrose (Figure 4c). These observations indicated carbohydrates as the characteristic markers during the occurrence of obstacles due to continuous cropping of *G. elata*.

#### 3.2.3 Correlation between the top 20 most abundant soil bacterial OTUs and the representative metabolites with different accumulation trends (by K-means class)

**Figure 5:**
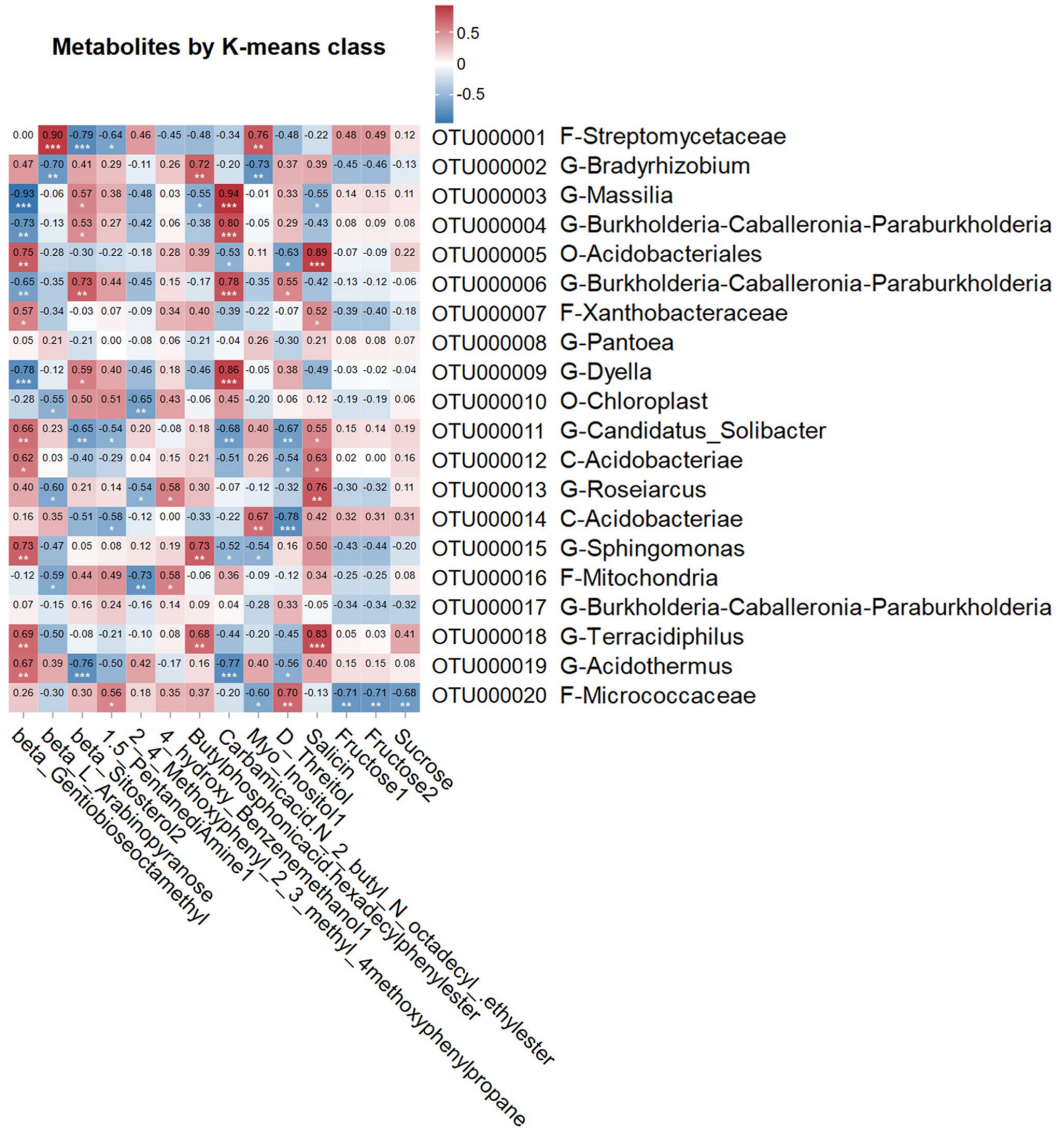
Correlation between the top 20 most abundant soil bacterial OTUs and the representative metabolites with different accumulation trends Between soil under continuous cropping obstacles of *G. elata*. Pearson coefficient was used to assess the correlation. The horizontal axis represents the top 20 most abundant soil metabolites (Figure 4B), and the vertical axis represents top 20 most abundant bacterial OTUs. The numbers indicate the correlation coefficient values. *, **, and *** indicate significant correlations at *p*-values between 0.01 and 0.05, 0.001 and 0.005, and less than 0.001, respectively.

We further analyzed the correlation between the metabolites revealed by K-means clustering and the top 20 most abandence soil bacterial OTUs. OTU000005 (Acidobacteriales), OTU000007 (Xanthobacteraceae), OTU000011 (*Candidatus_Solibacter*), OTU000012 (Acidobacteriae), OTU000015 (*Sphingomonas*), OTU000018 (*Terracidiphilus*), and OTU000019 (*Acidothermus*) showed a significant positive correlation with beta-Gentiobiose octamethyl (class 8, increased abundance in 1 and 2 Year; *p* < 0.05; Figure 5). Similarly, OTU000001 (Streptomycetaceae) was positively correlated with beta-L-arabinopyranose (class 10, low abundance from 0 Year to 3 Year; *p* < 0.05), OTU000003 (*Massilia*), OTU000004 and OTU000006 (*Burkholderia-Caballeronia-Paraburkholderia*), and OTU000009 (*Dyella*) with beta-sitosterol 2 (class 9, increased abundance from 0 to 3 Year; *p* < 0.05), and OTU000020 (Micrococcaceae) with 1,5-pentanediamine 1 (class 9; *p* < 0.05). Additionally, OTU000013 (*Roseiarcus*) and OTU000016 (Mitochondria) were positively correlated with 4-hydroxy-benzenemethanol 1 (class 6, abundance fluctuating from 0 Year to 3 Year; *p* < 0.05), and OTU000002 (*Bradyrhizobium*), OTU000015 (*Sphingomonas*), and OTU000018 (*Terracidiphilus*) with butylphosphonic acid and hexadecyl phenyl ester (class 2, increased abundance in 1 Year; *p* < 0.05). OTU000003 (*Massilia*), OTU000004 and OTU000006 (*Burkholderia-Caballeronia-Paraburkholderia*), and OTU000009 (*Dyella*) were positively correlated with carbamic acid, N-(2-butyl)-N-octadecyl-, ethyl ester (class 4, increased abundance in 0 Year; *p* < 0.05). OTU000001 (Streptomycetaceae) and OTU000014 (Acidobacteriae) were positively correlated with myo-inositol 1 (class 10; *p* < 0.05), OTU000006 (*Burkholderia-Caballeronia-Paraburkholderia*) and OTU000020 (*Terracidiphilus*) with D-threitol (class 1, increased abundance in 0 Year and 3 Year; *p* < 0.05), and OTU000005 (*Burkholderia-Caballeronia-Paraburkholderia*), OTU000007 (Xanthobacteraceae), OTU000011 (*Dyella*), OTU000012 (Acidobacteriae), OTU000013 (*Roseiarcus*), and OTU000018 (*Terracidiphilus*) with salicin (class 8; increased abundance in 1 Year and 2 Year, *p* < 0.05).

## 4 Discussion

Obstacles associated with continuous cropping significantly limit the stable and high production of *G. elata* tubers. The soil microbial and metabolic mechanisms underlying continuous cropping obstacles in *G. elata* and their correlation with microbial diversity remain unstudied. Therefore, the present study systematically analyzed and revealed the microbial and metabolic factors and their correlation to elucidate the mechanisms underlying this continuous cropping obstacle, laying the foundation for developing this resource.

### 4.1 Culture of *Gastrodia* disturb soil bacterial diversity

Continuous cropping obstacles are typically caused by soil nutrient deficiencies, accumulation of toxic metabolites, and disruption of microbial ecological balance (Ma et al., 2023). Soil replacement has efficiently mitigated the continuous cropping obstacles in culture of *Gastrodia*, indicating soil as a primary factor underlying their occurrence. Specifically, 0 Year (the year of *G. elata* harvest) and 1 Year (the year of continuous cropping obstacles occurrence) were identified as critical points of regulating obstacles to continuous cropping, exhibiting an increase in soil bacterial diversity compared with the uncultivated soil. Similarly, the continuous cropping cultivation of another medicinal plant, *Panax notoginseng*, also increased soil bacterial diversity (Bao et al., 2022); however, the onset of continuous cropping obstacles subsequently reduced the diversity (Tan et al., 2017). In contrast, the cultivation of *G. elata* led to an increase in soil bacterial diversity. Notably, the alpha diversity did not decline after the occurrence of continuous cropping obstacles (1 Year), indicating a difference in the underlying mechanism between *G. elata* and *P. notoginseng.* Typically, a difference in continuous cropping obstacles mechanism may largely be due to substantial disparities in soil nutrition and growth patterns between *P. notoginseng* and *G. elata.* The medicinal plant *P. notoginseng* is a typical species that performs photosynthesis and absorbs nutrients through its roots. On the contrary, *G. elata* is a specific species that relies solely on its symbiotic relationship with *Amillariella* sp. for food and sustenance, which *Amillariella* sp. is a macrofungal genera, some species within this genus can form symbiotic relationships with *G. elata* (Yuan et al., 2018). Research has confirmed that soil macrofungi significantly influence soil bacterial diversity. Interestingly, various fungal species, for instance, species like *Morchella esculenta* (Duan et al., 2023), *Dictyophora indusiata* (Duan et al., 2023), and *Leucocalocybe mongolica* (Duan et al., 2022) have been proven to alter soil bacterial communities. Thus, we assume that *Amillariella* sp. is one of the key factors regulating the soil bacterial diversity in *G. elata* fields and influencing the occurrence of continuous cropping obstacles. Therefore, focusing future investigations on how *Armillaria* alone influences soil microbial diversity, soil metabolism regulation, and soil chemical composition regulation, and comparing these findings with *G. elata* cultivation, will provide new insights into the mechanisms behind this continuous cropping obstacle.

### 4.2 Continuous cropping of *Gastrodia elata* leads to differences in soil bacterial functions

We identified soil bacteria related to the continuous cropping obstacle of *G. elata* based on FAPROTAX annotation. We found that the abundance of bacteria associated with soil methane metabolism decreased after *G. elata* cultivation (0 Year). Typically, the microbial communities in the soil produce methane as a part of anaerobic respiration; hence, this class of bacteria is found substantially enriched in low-oxygen environments, such as wetlands and rice paddies (Nazaries et al., 2013). In other words, the low abundance of methane-producing bacteria after *G. elata* cultivation (0 Year) indicates high oxygen content in the soil, potentially reducing the abundance of anaerobic bacteria and disrupting the soil microbial balance, resulting in continuous cropping obstacles. Specifically, in the year of continuous cropping obstacles (1 Year), the abundance of bacterial communities related to methanol oxidation was significantly high, while the abundance of methane-metabolizing bacterial communities was low. This phenomenonis likely because methanol oxidation produces methane monooxygenase (MMO) in the soil, further oxidizing methane (Cai et al., 2016). At the same time, this phenomenon may be related to our previous hypothesis that soil oxygen levels increase after *G. elata* cultivation. The above suggests that *G. elata* cultivation may lead to an increase in soil oxygen content, which could subsequently oxidize methane. This change may regulate soil microbial diversity, ultimately contributing to the occurrence of continuous cropping obstacles in *G. elata*. This is an interesting hypothesis that warrants further investigation in the future.

Furthermore, we found that sulfur metabolism-related bacteria were more abundant in the 0 Year group among all the groups. Sulfur is an essential micronutrient that promotes the formation of disulfide bonds in proteins, amino acids, vitamins, and cofactors, facilitating plant growth (Narayan et al., 2023). However, *G. elata* cannot absorb sulfur through its roots, and therefore we suppose the high intensity of sulfur metabolism due to *G. elata* cultivation may lead to sulfur accumulation, thereby disrupting soil bacterial diversity and causing continuous cropping obstacles. This observation suggests that the concentration of soil ions, such as sulfides, in the soil might also be a crucial factor responsible for the continuous cropping obstacle. A study on the macro fungi of *Leucocalocybe mongolica* found that it can regulate soil ion abundance, thereby affecting plant growth (Duan et al., 2022). Given that *G. elata* cultivation also involves interactions between a macro fungi and soil, we believe there is a significant likelihood that soil ion factors are regulated by *Armillaria* released during the cultivation of *G. elata*. However, further studies should confirm this.

### 4.3 Metabolic factors causing *Gastrodia elata* continuous cropping obstacles

Generally, soil metabolic processes reflect the activity of soil microbes (Withers et al., 2020). Therefore, metabolic factors during the occurrence of *G. elata* continuous cropping obstacle indicate the key regulatory factors of the obstacle. The present study found that 4-hydroxy-benzenemethanol 1 was actively expressed during the *G. elata* continuous cropping obstacle (1 Year, Figure 4b and Table 1) and therefore identified as a potential associated factor. Research has proven that 4-hydroxy-benzenemethanol 1 is a signature metabolite and as an active medicinal component of *G. elata* (Zhang et al., 2020) and is also metabolized by macrofungi (Gressler et al., 2021). However, the present study’s observations suggest that the metabolite is may related to continuous cropping obstacles and not directly linked to *G. elata* growth. In addition, we discovered many carbohydrates (beta-gentiobiose octamethyl, beta-L-arabinopyranose, D-threitol, salicin, fructose 1, fructose 2, and sucrose; Figure 4c) associated with the *G. elata* continuous cropping obstacle. Some studies on soil-microbe interactions in the soils detected increased carbohydrate metabolites in soil samples with intense and frequent microbial activities (Duan et al., 2022; Duan et al., 2023). Thus, we conclude that microbial activities in the soil and associated metabolic processes are key factors in the occurrence of obstacles after *G. elata* continuous cropping.

### 4.4 Association among the key soil bacteria, metabolites, and *Gastrodia elata* continuous cropping obstacles

Finally, we found a link between key soil bacteria and metabolites that may regulate the occurrence of continuous cropping obstacles in *G. elata* based on Pearson’s correlation analysis. OTU000003 (*Massilia*), OTU000004 (*Burkholderia-Caballeronia-Paraburkholderia*), and OTU000009 (*Dyella*) were found to positively regulate carbamic acid, N-(2-butyl) -N-octadecyl-, ethyl ester, a soil metabolite produced during the cultivation of *G. elata*, which may be the initial factor triggering continuous cropping obstacles.

The abundance of this metabolite was low in control soil, significantly enriched in the 0 Year group, and low in the 1 Year group. Based on these observations, we speculate that the enrichment of this metabolite is due to the growth of three soil bacteria, *Massilia*, *Burkholderia-Caballeronia-Paraburkholderia*, and *Dyella*, accompanying the growth of *G. elata*. In the 1 Year soil, this metabolite probably regulated the balance of soil bacteria and the growth of *G. elata*, leading to continuous cropping obstacles. This due to metabolite of Carbamic is known to possess herbicide, insecticide, and fungicide activities (Li et al., 2010; Rendler, 2016; George et al., 1954), and it inhibits the growth of plants, insects, and microorganisms (bacteria, fungi, and archaea) in the soil. Interestingly, the soil bacterium *Massilia* can degrade herbicides (Lee et al., 2017). Thus, the positive correlation detected in this study between the abundance of *Massilia* and the accumulation of carbamic acid ester suggests the stimulation by increased levels of this metabolite. Additionally, *Burkholderia-Caballeronia-Paraburkholderia* and *Dyella* can degrade toxic soil metabolites. For instance, the *Burkholderia-Caballeronia-Paraburkholderia* strain B36 was proven to degrade toxic ginsenosides in soil and antagonize soil-borne pathogen *Ilyonectria destructans* (Luo et al., 2021), while *Dyella* has soil biphenyl-degrading capabilities (Li et al., 2009). Therefore, the abundance of *Massilia*, *Burkholderia-Caballeronia-Paraburkholderia*, and *Dyella* during the continuous cropping obstacles of *G. elata* appears to be a response to the invasion of extreme metabolites, which acts by regulating soil bacterial communities. Future studies should investigate the sources of these metabolites like carbamic acid, N-(2-butyl)-N-octadecyl-, ethyl ester to confirm whether they are the primary factors responsible for these continuous cropping obstacles.

## 5 Conclusion

This study has shed light on the soil bacteria and metabolites underlying the continuous cropping obstacles of *Gastrodia elata*. The identified 4-hydroxy-benzenemethanol and specific carbohydrates offer important clues. We found that the cultivation of *Gastrodia elata* affects soil bacterial diversity and functions. Key soil bacteria like *Massilia, Burkholderia-Caballeronia-Paraburkholderia*, and *Dyella* are correlated with certain metabolites. Future research should explore the origin of these metabolites and assess soil ionomes. Practical measures could include monitoring soil factors and implementing soil management practices for sustainable cultivation.

## Data Availability

The datasets presented in this study can be found in online repositories. The names of the repository/repositories and accession number(s) can be found at: https://www.ncbi.nlm.nih.gov/, PRJNA1142202.

## Footnotes

^1^ http://CRAN.R-project.org/package=vegan

^2^https://www.omicshare.com/tools (Accessed May 21, 2024).

^3^ https://cran.rproject.org/src/contrib/Archive/psych/
