# Supplementary figures and images for: Unraveling the key mechanisms of Gastrodia elata continuous cropping obstacles: soil bacteria Massilia, Burkholderia-Caballeronia-Paraburkholderia, and Dyella along with soil metabolites 4-hydroxy-benzenemethanol and N-(2-butyl)-N-octadecyl-, ethyl ester as crucial indicators
Source: Front Microbiol. 2024 Oct 28;15:1478330. doi: 10.3389/fmicb.2024.1478330 (PMC11550952; doi:10.3389/fmicb.2024.1478330)

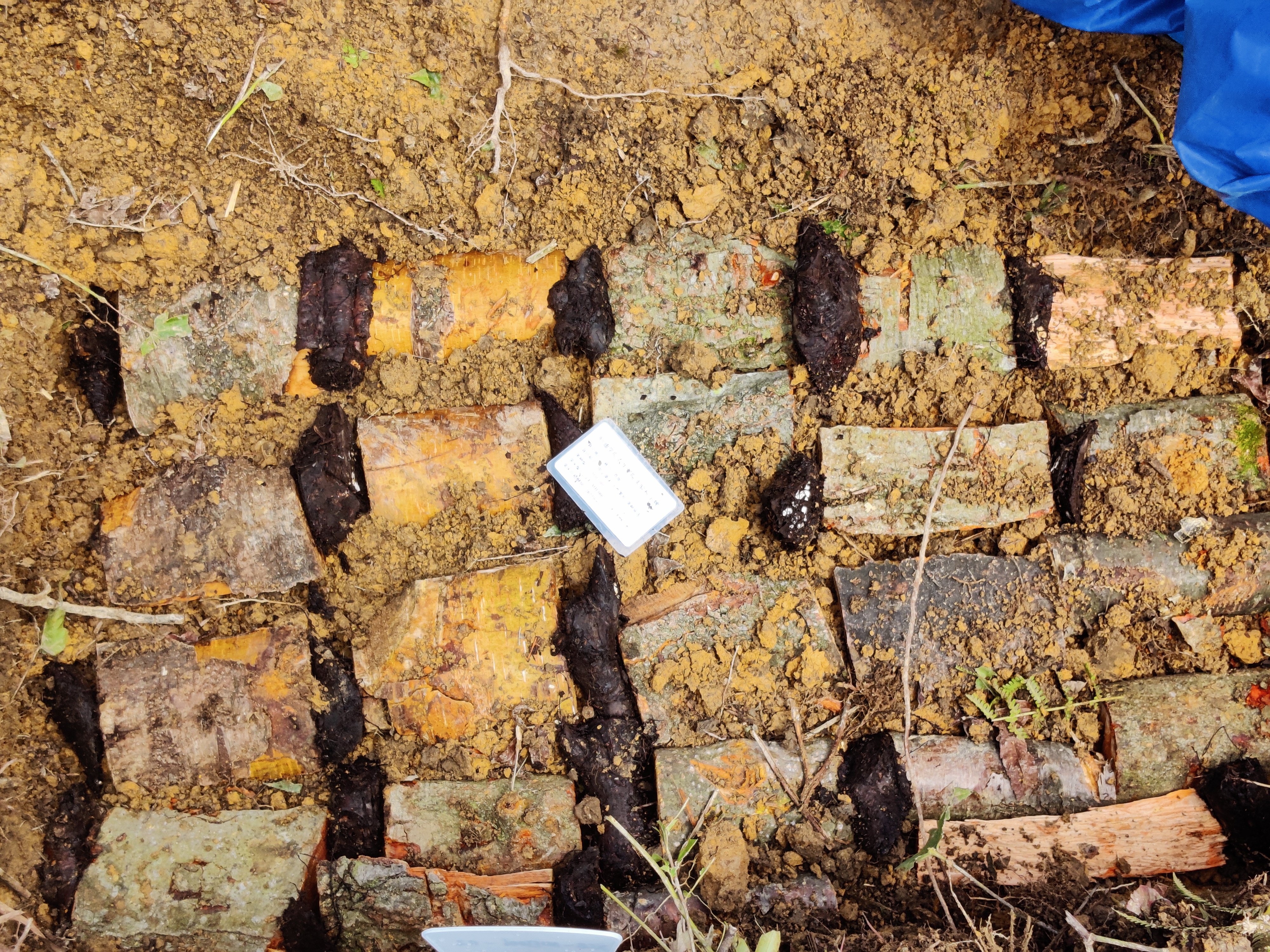

Supplement: Supplementary file 1 [file Image_1.JPEG]
